# Local Application of Mineral-Coated Microparticles Loaded With VEGF and BMP-2 Induces the Healing of Murine Atrophic Non-Unions

**DOI:** 10.3389/fbioe.2021.809397

**Published:** 2022-01-11

**Authors:** M. Orth, T. Fritz, J. Stutz, C. Scheuer, B. Ganse, Y. Bullinger, J. S. Lee, W. L. Murphy, M. W. Laschke, M. D. Menger, T. Pohlemann

**Affiliations:** ^1^ Department of Trauma, Hand and Reconstructive Surgery, Saarland University, Homburg, Germany; ^2^ Institute for Clinical and Experimental Surgery, Saarland University, Homburg, Germany; ^3^ Werner Siemens Endowed Chair of Innovative Implant Development (Fracture Healing), Saarland University, Homburg, Germany; ^4^ Department of Biomedical Engineering, University of Wisconsin-Madison, Madison, WI, United States

**Keywords:** non-union, mineral-coated microparticles, VEGF, BMP-2, bone healing, fracture

## Abstract

Deficient angiogenesis and disturbed osteogenesis are key factors for the development of nonunions. Mineral-coated microparticles (MCM) represent a sophisticated carrier system for the delivery of vascular endothelial growth factor (VEGF) and bone morphogenetic protein (BMP)-2. In this study, we investigated whether a combination of VEGF- and BMP-2-loaded MCM (MCM + VB) with a ratio of 1:2 improves bone repair in non-unions. For this purpose, we applied MCM + VB or unloaded MCM in a murine non-union model and studied the process of bone healing by means of radiological, biomechanical, histomorphometric, immunohistochemical and Western blot techniques after 14 and 70 days. MCM-free non-unions served as controls. Bone defects treated with MCM + VB exhibited osseous bridging, an improved biomechanical stiffness, an increased bone volume within the callus including ongoing mineralization, increased vascularization, and a histologically larger total periosteal callus area consisting predominantly of osseous tissue when compared to defects of the other groups. Western blot analyses on day 14 revealed a higher expression of osteoprotegerin (OPG) and vice versa reduced expression of receptor activator of NF-κB ligand (RANKL) in bone defects treated with MCM + VB. On day 70, these defects exhibited an increased expression of erythropoietin (EPO), EPO-receptor and BMP-4. These findings indicate that the use of MCM for spatiotemporal controlled delivery of VEGF and BMP-2 shows great potential to improve bone healing in atrophic non-unions by promoting angiogenesis and osteogenesis as well as reducing early osteoclast activity.

## Introduction

Despite growing knowledge about the process of bone healing, 5–10% of all fractures still fail to heal (Buza and Einhorn, 2016). The resulting burden for patients and the socioeconomic consequences of non-unions are a major clinical problem (Bishop et al., 2012; Buza and Einhorn, 2016). Key factors for delayed fracture healing and development of non-unions are known to be a deficient angiogenesis and disturbed osteogenesis (Kanczler and Oreffo, 2008). In clinical practice, the gold-standard for the treatment of non-unions is still the transplantation of autologous bone tissue. However, this procedure does not guarantee adequate bone healing and bears the risk of a number of complications, such as donor-site morbidity, infections and additional pain (Pelissier et al., 2003; Yoneda et al., 2005).

To prevent patients from such complications and to improve the fusion rate of not healing fractures, artificial bone substitutes have been developed. Lately, these materials have been optimized by combining osteoconductive and osteoinductive properties in order to support natural bone growth and to induce new bone formation (Fillingham and Jacobs, 2016). Among others, mineral-coated microparticles (MCM) were developed to enable a biomimetic, tunable growth factor delivery (Yu et al., 2014). MCM are biocompatible, can easily be applied to a fracture site by injection, and allow for a controllable binding and spatiotemporal controlled release of growth factors (Yu et al., 2014; Yu et al., 2017). Furthermore, MCM have been analyzed in detail regarding binding and release kinetics for vascular endothelial growth factor (VEGF) and bone morphogenetic protein (BMP)-2 (Yu et al., 2014).

VEGF is a well-known key regulator of physiological angiogenesis during embryogenesis and skeletal growth (Ferrara et al., 2003), whereas BMP-2 promotes differentiation of mesenchymal stem cells and osteoprogenitor cells into osteoblasts and thereby acts osteogenic (Cheng et al., 2003). The application of these growth factors using MCM as a carrier system previously demonstrated improved osseous bone formation in non-unions (Orth et al., 2017; Orth et al., 2019).

Based on these promising previous findings, we herein hypothesized that the combined application of MCM loaded with VEGF and BMP-2 may improve bone healing in atrophic non-unions. To test our hypothesis, we administered MCM loaded with VEGF and BMP-2 with a ratio of 1:2 in a well-established murine non-union model and studied the healing process by means of radiological, biomechanical, histomorphometric and Western blot techniques throughout an observation period of 70 days.

## Materials and Methods

### Preparation of MCM

MCM were prepared by incubating 100 mg hydroxyapatite particles (Biotal LTD., Derbyshire, United Kingdom) in 50 ml modified simulated body fluid (mSBF) at 37°C for 7 days, as described previously (Yu et al., 2014; Orth et al., 2017; Orth et al., 2019). The mSBF contains the similar ionic constituents to human plasma with doubled concentration of calcium and phosphate ions to promote mineral growth. It was prepared by adding the following reagents (Thermo Fisher Scientific, Waltham, United States) into deionized water in the order shown: 141 mM NaCl, 4.0 mM KCl, 0.5 mM MgSO_4_, 1.0 mM MgCl_2_, 4.2 mM NaHCO_3_, 20.0 mM HEPES, 5.0 mM CaCl_2_, and 2.0 mM KH_2_PO_4_, and the pH was adjusted to 6.80. During the preparation process, the mSBF was changed daily to maintain consistent ion concentrations for mineral coating growth on the particles. After the incubation period, the coated microparticles were rinsed with deionized water and lyophilized. Morphological and elemental analyses were carried out before and after coating using LEO 1530 field emission scanning microscopy (FE-SEM; Zeiss, Oberkochen, Germany) and energy dispersive spectrometer (EDS) equipped on FE-SEM, respectively. Transmission FT-IR was performed by a Nicolet iS50R FT-IR spectrometer (Thermo Fisher Scientific) to characterize chemical composition, and ImageJ software (National Institutes of Health, Bethesda, United States) was used to determine the size of hydroxyapatite particles and MCM based on SEM images.

### Binding of VEGF and BMP-2 to MCM

MCM were either loaded with recombinant human (rh) VEGF or rhBMP-2 (ImmunoTools GmbH, Friesoythe, Germany). For loading with VEGF, 1 mg MCM was incubated in 1 ml PBS containing 21.7 µg VEGF at 37°C for 4 h. This resulted in a VEGF concentration of 10 μg/mg MCM for the *in vivo* experiments (Orth et al., 2019). The applied amount of VEGF was chosen upon previous studies assessing the effect of VEGF on bone healing in rodents (Street et al., 2002; Ogilvie et al., 2012; Amirian et al., 2015; Orth et al., 2019). For loading with BMP-2, 1 mg MCM was incubated in 1 ml PBS containing 34 µg BMP-2 at 37°C for 4 h. This resulted in a BMP-2 concentration of 20 μg/mg MCM for the *in vivo* experiments (Yu et al., 2014; Orth et al., 2017). After binding either VEGF or BMP-2 on separate microparticles, the loaded MCM were centrifuged for 3 min and then washed once with PBS. For the application *in vivo*, 2 mg MCM in total, of which each microparticle was either loaded with VEGF or BMP-2, was used.

### Animals

A total of 72 CD-1 mice with a body weight of 30 ± 5 g and an age of 9–14 weeks were used. The animals were bred at the Institute for Clinical and Experimental Surgery, Saarland University, Germany, kept at a regular light and dark cycle and had free access to tap water and standard pellet food (Altromin, Lage, Germany). The study was conducted in accordance with the German legislation on protection of animals and the NIH Guidelines for the Care and Use of Laboratory Animals and was approved by the local governmental animal protection committee (permission number: 53/2013).

### Surgical Procedure

For the present study a well-established femoral atrophic non-union model was used, as described previously in detail (Garcia et al., 2008). For the surgical procedure, the mice were anesthetized by an intraperitoneal injection of ketamine (75 mg/kg body weight; Pharmacia, Erlangen, Germany) and xylazine 2% (25 mg/kg body weight; Bayer, Leverkusen, Germany). Briefly, an incision medial to the patella was performed at the right knee and the patella was dislocated laterally. After exposing the intercondylar notch, a hole was drilled between the femoral condyles to insert a distally flattened pin through the intramedullary canal. After implantation of the pin, the diaphysis of the femur was exposed and an approximately 6 mm clip was inserted into the femur. Subsequently, an osteotomy with a gap size of 1.8 mm was created between the two brackets of the metallic clip. Inserting the clip before creation of the gap guaranteed that the gap size was maintained. Finally, the periosteum was stripped 2 mm proximally and distally of the gap.

Animals of the group MCM + VB (n = 24) received 1 mg MCM loaded with a total of 10 µg VEGF and 1 mg MCM loaded with a total of 20 µg BMP-2 by direct injection into the osteotomy gap without any further carrier material. Of interest, we could previously show that MCM without physical support do not dissolve and can still be detected at the site of implantation in our non-union model even after 70 days (Orth et al., 2017). Animals of the group MCM (n = 24) received 2 mg growth factor-free MCM under the same conditions. The animals of the control group (n = 24) received no MCM, leaving the osteotomy gap empty. Finally, wound closure completed the surgical procedure.

Animals were sacrificed on day 14 (n = 12 each group) or 70 (n = 12 each group) postoperatively. Directly prior to sacrifice, X-rays of the operated femurs were taken to exclude secondary dislocation of the metallic implants. Femurs were harvested and used for further analyses.

### Biomechanical Analysis

After removal of the pin and the clip as well as the surrounding soft tissue, 3-point-bending stiffness of the osteotomized femurs was measured (*n* = 8 each group; Mini-Zwick Z 2.5, Zwick, Ulm, Germany), as described previously (Orth et al., 2017; Orth et al., 2019). Using this non-destructive approach for biomechanical analyses, the femurs could also be used for subsequent micro-computed tomography (µCT) and histological investigations, resulting in a marked reduction of required animals. To account for differences in bone strength of individual animals, the non-osteotomized left femora were also analyzed and served as internal controls.

### µCT

The femurs (n = 8 each group) were scanned (Skyscan 1176, Bruker, Billerica, United States) at a spatial resolution of 9 μm with a standardized setup, as described previously (Orth et al., 2017; Orth et al., 2019). To express gray values as mineral content (bone mineral density; BMD), calcium hydroxyapatite (CaHA) phantom rods with known BMD values were used for calibration. On each transversal slide the region of interest (ROI) was contoured manually defining exclusively novel bone and excluding original cortical bone and the applied MCM. The ROI was processed with a threshold procedure (CTAnalyzer, Bruker), which allowed for differentiation between highly and poorly mineralized bone. The thresholds to distinguish between highly and poorly mineralized bone were based on visual inspection of the images, qualitative comparison with histological sections and other studies investigating bone repair and callus tissue by µCT (Isaksson et al., 2009; Morgan et al., 2009; Bosemark et al., 2013). A BMD with more than 0.642 g/cm^3^, resulting in gray values of 98–255, was defined as high mineralized bone. Low mineralized bone was assumed to have a BMD value between 0.410 g/cm^3^ and 0.642 g/cm^3^, resulting in gray values of 68–97. A BMD with more than 0.41 g/cm^3^, resulting in gray values of 68–255, was defined as total mineralized bone.

The following radiological parameters were calculated from the callus region of interest for each specimen: High mineralized bone volume (BV_high_; mm^3^), low mineralized bone volume (BV_low_; mm^3^) and average bone mineral density for mineralized bone (BMD; g hydroxyapatite(HA)/cm^3^). The BMD was calculated by using the voxels within the aforementioned thresholds.

### Histology and Histomorphometry

After biomechanical testing and µCT analyses, bones were fixed in 4% phosphate-buffered formalin for 24 h and decalcified in ethylenediaminetetraacetic acid (EDTA) solution for several weeks. After embedding decalcified bones in paraffin, longitudinal sections with a thickness of 5 µm were stained with Safranin-O (n = 8 each group). At a magnification of ×12.5 (Olympus BX60 Microscope, Olympus, Shinjuku, Japan; Zeiss Axio Cam and Axio Vision 3.1, Zeiss, Jena, Germany) structural indices were calculated based on the recommendations of Gerstenfeld et al. (Gerstenfeld et al., 2005). For histomorphometric evaluation the following parameters were measured: 1) total periosteal callus area, 2) bone callus area, 3) cartilaginous callus area and 4) fibrous callus area. The total periosteal callus area was defined as all osseous, cartilaginous and fibrous callus tissue outside of the cortices. Pre-existing cortical bone and endosteal callus formation were excluded as it may have been affected by the intramedullary pin. Each area was marked and calculated using the ImageJ Analysis System.

Additionally, a scoring system was used to evaluate the quality of gap bridging on both postoperative time points, as described previously (Orth et al., 2017). This scoring system allowed a maximum of four points that indicated complete osseous bridging of the non-union, while zero points indicated the absence of osseous or cartilaginous bridging of the gap.

### Immunohistochemistry

Longitudinal sections of the femora were co-stained with a monoclonal rat anti-mouse antibody against CD31 (1:100; Abcam, Cambridge, United Kingdom), while a goat anti-rat IgG antibody (Invitrogen, Waltham, United States) served as secondary antibody. Cell nuclei were stained with Hoechst 33342 (2 μg/ml; Sigma-Aldrich, Taufkirchen, Germany). The number of CD31-positive (C31^+^) microvessels in animals of the groups control, MCM and MCM + VB at 14 and 70 days postoperatively was counted at a magnification of ×400 (Olympus BX60 microscope) in the central healing zone of the periosteal callus using two high-power fields (HPF) per specimen.

### Western Blot

Protein expression within the callus tissue was determined by Western blot analyses, including the expression of the osteoclast markers osteoprotegerin (OPG) and receptor activator of NF-κB ligand (RANKL), the angiogenic markers erythropoietin (EPO) and EPO-receptor (EPO-R), the osteogenic marker BMP-4 and the proliferation marker proliferating cell nuclear antigen (PCNA). After harvesting callus tissue on post-operative days 14 (n = 4 each group) and 70 (n = 4 each group), tissue samples were transferred in lysis buffer and stored at −80°C. After saving the whole protein fraction, proteins were separated and transferred to membranes by standard protocols and probed using anti-OPG (Bioss (Biozol), Eching, Germany), anti-RANKL (Abcam), anti-EPO (Santa Cruz Biotechnology, Heidelberg, Germany), anti-EPO-R (Santa Cruz Biotechnology), anti-BMP-4 (Santa Cruz Biotechnology) and anti-PCNA (Dako (Agilent), Hamburg, Germany) antibodies. All antibodies were incubated overnight in concentration of 1:30 at 4°C and afterwards for 4 h in a concentration of 1:300 at room temperature. The appropriate peroxidase-conjugated anti-IgG antibodies served as secondary antibodies (1:1,000 for 1.5 h, Dako (Agilent)). Protein expression was visualized by means of luminol-enhanced chemiluminescence after exposure of the membrane to the Intas ECL Chemocam Imager (Intas Science Imaging Instrument GmbH, Göttingen, Germany). To correct for unequal loading, signals were normalized to β-actin signals (1:5,000, Sigma-Aldrich).

### Statistics

All data are given as means ± standard error of the mean (SEM). First, data were tested for normal distribution and equal variance. In case of parametric data, comparisons between two experimental groups were performed by an unpaired Student’s *t*-test, while analyses of three groups were performed by one-way ANOVA, followed by the Student-Newman-Keuls test for all pairwise comparisons, including the correction of the α-error according to Bonferroni probabilities to compensate for multiple comparisons. In case of non-parametric data, comparisons between two experimental groups were performed by a Mann-Whitney Rank Sum Test, while analyses of three groups were performed by one-way ANOVA on Ranks, followed by a Student-Newman-Keuls test for all pairwise comparisons, which also included the correction of the α-error according to Bonferroni probabilities. The statistical analyses were performed using the SigmaPlot software 13.0 (Systat Software GmbH, Erkrath, Germany). A *p*-value < 0.05 was considered to indicate significant differences.

## Results

### MCM Fabrication and Characterization

**FIGURE 1:**
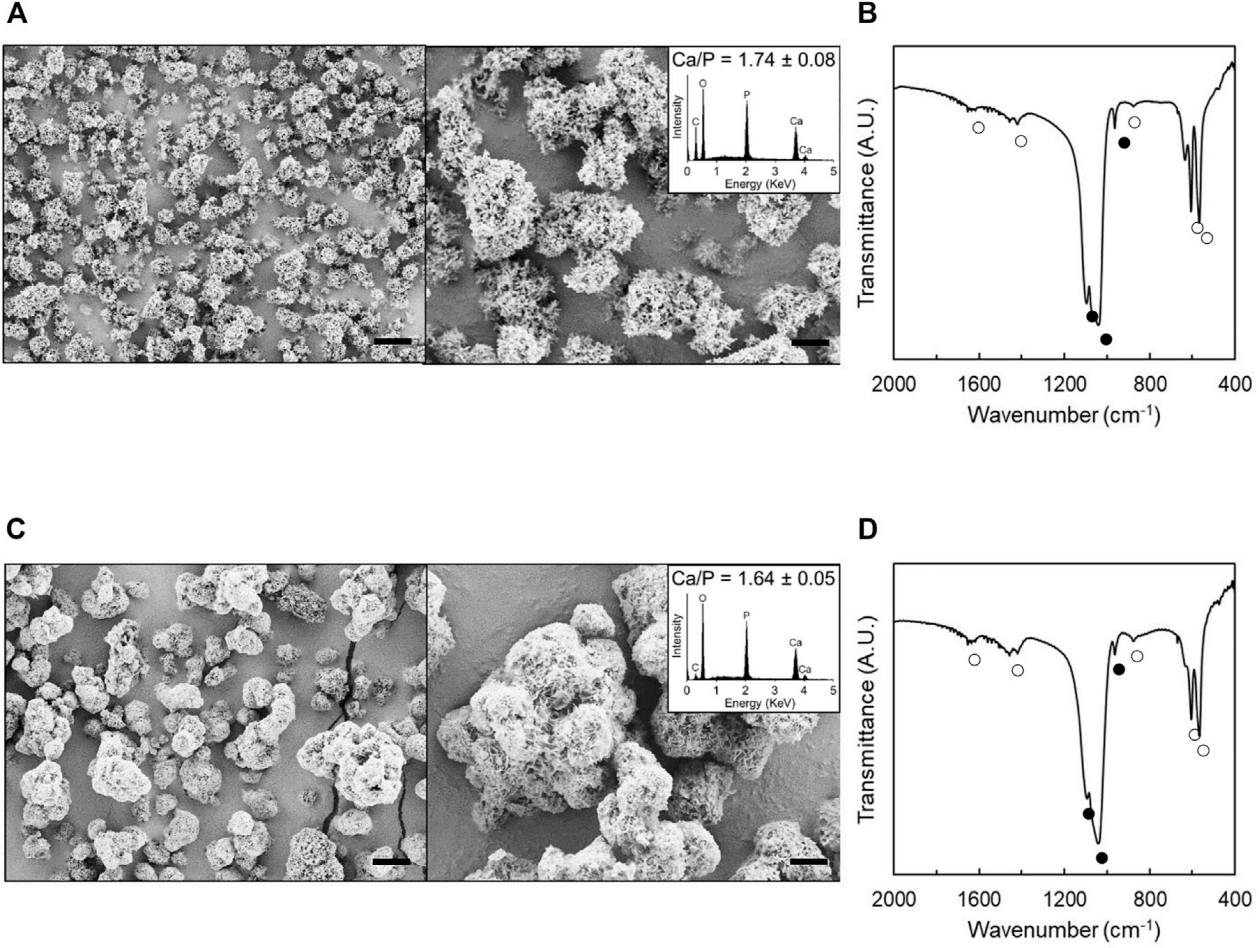
Morphological and compositional analyses of hydroxyapatite particles and MCM. **A, C:** SEM images of hydroxyapatite particles **(A)** and MCM **(C)**. Insets show their EDS spectrum. **B, D:** FT-IR spectra of hydroxyapatite particles **(B)** and MCM **(D)**. ● and ○ denote the peaks associated with phosphate and carbonate, respectively. Scale bars: 5 µm (left images) and 2 µm (right images).

We created a mineral coating on hydroxyapatite particles by incubation in mSBF. The mineral coating grown on hydroxyapatite particles had a plate-like structure with nanometer scale pores (Figure 1A,C). The analysis of SEM images revealed that MCM have an average diameter of 5.5 ± 2.6 µm, which is about 2 µm larger than that of hydroxyapatite particles before coating (3.4 ± 1.0 µm). It is speculated that the mineral coating is ∼1 µm in thickness. This result is in agreement with our previous observation by SEM (Yu et al., 2017). The elemental analysis by EDS showed that the calcium to phosphorus ratio (Ca/P) of hydroxyapatite particles (1.74 ± 0.08) is similar to that of MCM (1.64 ± 0.05) (insets, Figure 1A,C). FT-IR spectra of uncoated hydroxyapatite particles and MCM showed characteristic peaks associated with phosphate (560–610, 930–1150 cm^−1^) and carbonate (850–890, 1410–1480, 1640–1700 cm^−1^) (Figure 1B,D) (Lee et al., 2010). The intensity of carbonate peaks from MCM was slightly more prominent compared to those of uncoated hydroxyapatite particles. Taken together, these results demonstrate that the coating of the MCM is plate-like, nanoporous, carbonate-substituted hydroxyapatite.

### X-Ray Analysis

**FIGURE 2:**
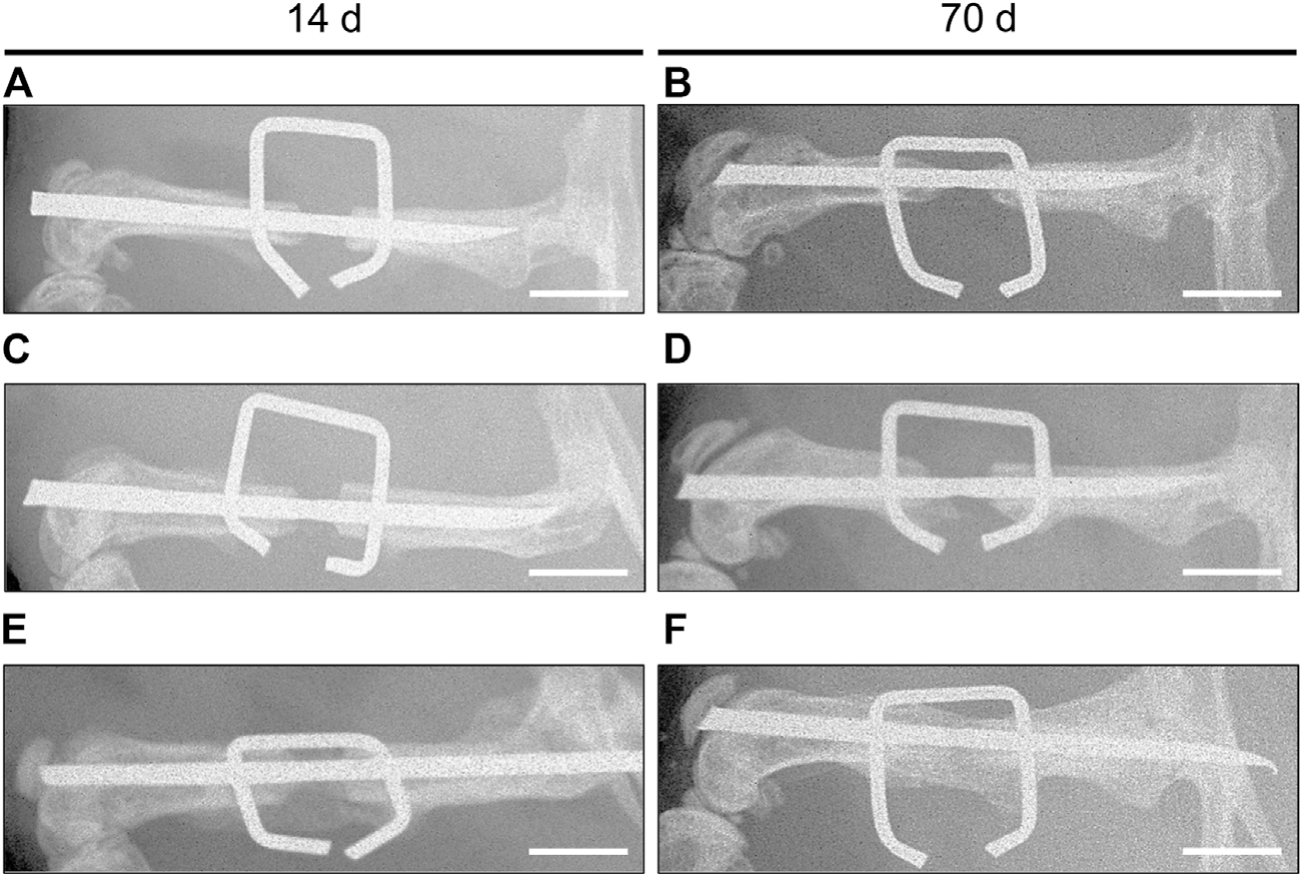
X-ray analysis of mouse femurs. **A-F**: X-rays of osteotomized mouse femurs with a segmental defect of 1.8 mm stabilized by the “pin-clip” technique 14 days **(A, C, E)** and 70 days **(B, D, F)** after osteotomy. Images of control **(A, B)** and MCM **(C, D)** exhibit no osseous bridging with a large persisting gap between the two bone fragments, indicating non-union formation, while images of MCM + VB **(E, F)** show callus formation after 14 days **(E)** and fully osseous bridging after 70 days **(F)**. Note that images of MCM + VB at 70 days **(F)** show signs of remodeling between the brackets of the clip, while images of controls at 70 days **(B)** exhibit that adjoining bone fragments narrowed towards the osteotomy gap as a typical sign for atrophic non-union formation. Scale bars: 2 mm.

X-rays of animals of the control and MCM group showed no osseous bridging with a large persisting gap between the two bone fragments (Figure 2A–D), whereas X-rays taken prior to sacrificing showed a radiopaque callus formation after 14 days and fully osseous bridging after 70 days in animals of the MCM + VB group (Figure 2E,F). Of interest, X-rays of animals of the MCM + VB group showed a dense and streamlined callus as radiological signs of bone remodeling and indicator of progressive bone healing at 70 days (Figure 2F). In contrast X-rays of the control group demonstrated that the form of the adjoining bone fragments narrowed towards the osteotomy gap as a typical sign for atrophic non-union formation (Figure 2B).

### Biomechanical Analysis

**FIGURE 3:**
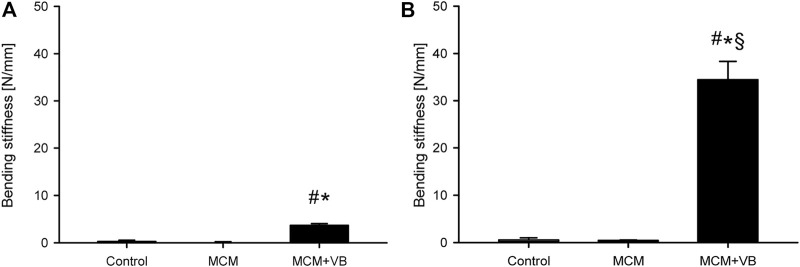
Biomechanical analysis of mouse femurs. **A, B:** Bending stiffness of control (*n* = 8), MCM (n = 8) and MCM + VB (n = 8) femurs 14 days **(A)** and 70 days **(B)** after osteotomy. Mean ± SEM; ^#^ *p* < 0.05 vs control; ^*^ *p* < 0.05 vs MCM; ^§^ *p* < 0.05 vs MCM + VB at day 14.

Femurs of the MCM and control group presented practically with no bending stiffness at both time points, which indicates non-union formation in these groups (Figure 3). In contrast, femurs of the MCM + VB group exhibited a significantly higher bending stiffness when compared to femurs of the MCM and control group at 14 and 70 days postoperatively (Figure 3A,B). Moreover, femurs of the MCM + VB group showed a significant increase of bending stiffness over the study time, indicating bone healing of the non-unions (Figure 3B). At 70 days after surgery, bending stiffness of femurs in the MCM + VB group reached approximately 65.8% of that measured for unfractured femurs.

### µCT Analysis

**FIGURE 4:**
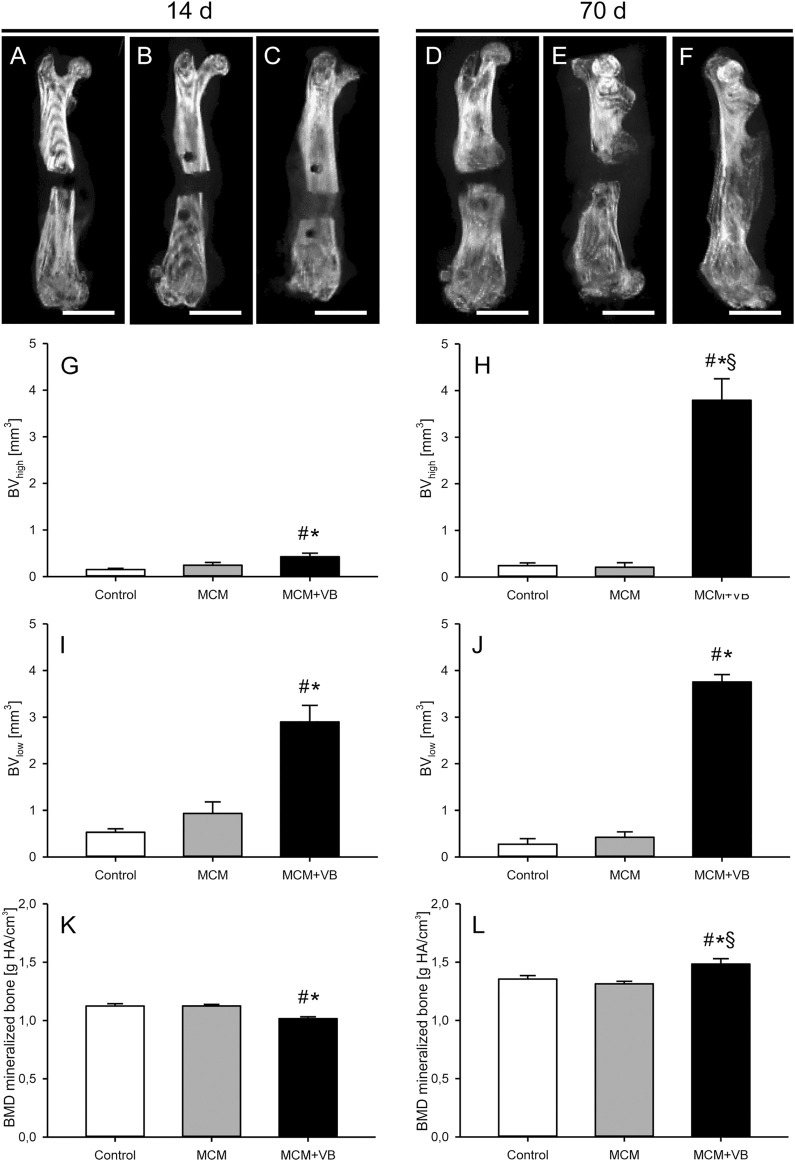
µCT analysis of mouse femurs. **A-F:** µCT images of femurs at 14 days **(A-C)** and 70 days **(D-F)** after surgery of control **(A, D)**, MCM **(B, E)** and MCM + VB **(C, F)** animals. Scale bars: 2 mm. **G, H:** Volume of high mineralized bone (BV_high_) at 14 days **(G)** and 70 days **(H)** after osteotomy within the callus of control (n = 8), MCM (n = 8) and MCM + VB (n = 8) femurs. **I, J**: Volume of low mineralized bone (BV_low_) at 14 days **(I)** and 70 days **(J)** after osteotomy within the callus of control (n = 8), MCM (n = 8) and MCM + VB (n = 8) femurs. **K, L**: BMD of total mineralized bone volume at 14 days **(K)** and 70 days **(L)** after osteotomy within the callus of control (n = 8), MCM (n = 8) and MCM + VB (n = 8) femurs. Mean ± SEM; ^#^ *p* < 0.05 vs control; ^*^ *p* < 0.05 vs MCM. ^§^ *p* < 0.05 vs MCM + VB at day 14.

µCT analyses revealed a significantly higher formation of high and low mineralized bone tissue on day 14 and 70 in the osteotomy gaps of MCM + VB animals when compared to those of MCM and control animals (Figure 4A–J). Of interest, the fraction of high mineralized bone in animals of the MCM + VB group at 14 days was still low and significantly increased over time (Figure 4G,H), whereas the fraction of low mineralized bone in these animals did not increase in the same period (Figure 4I,J). As a sign for mineralization and, thus, remodeling of the callus, the BMD increased in animals of the MCM + VB group during the study period (Figure 4K,L).

### Histomorphometric Analysis

**FIGURE 5:**
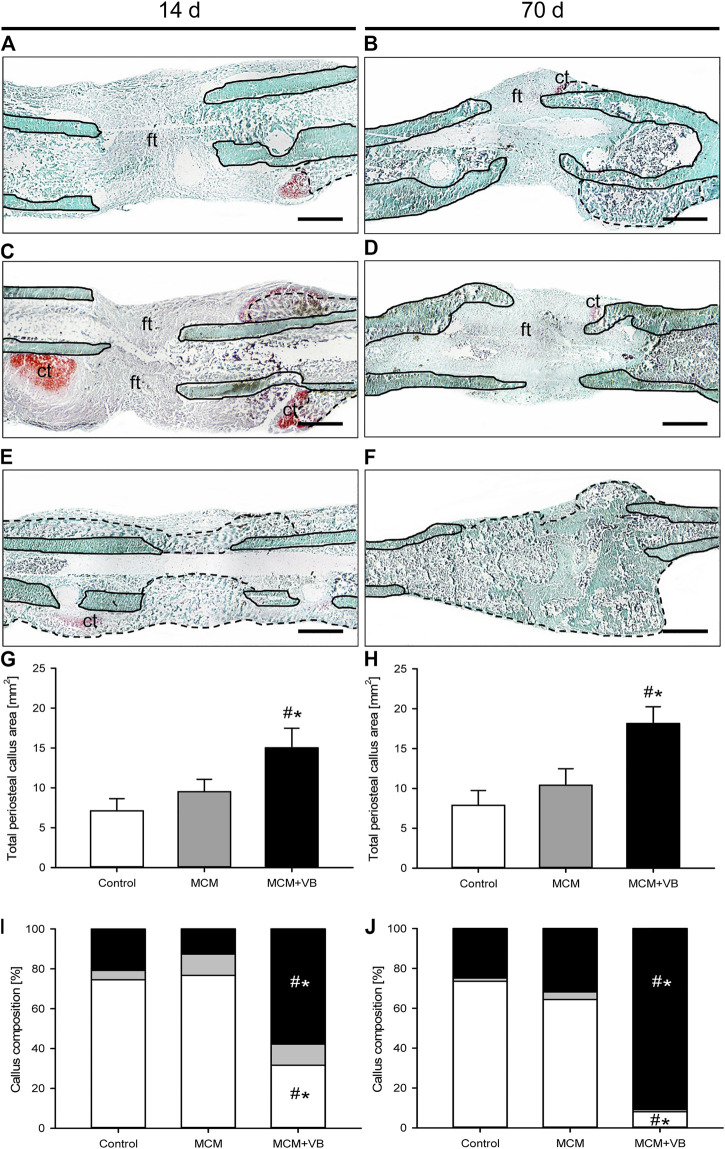
Histomorphometric analysis of mouse femurs. **A-F:** Representative histological images of Safranin-O-stained femurs at 14 days **(A, C, E)** and 70 days **(B, D, F)** after osteotomy of control **(A, B)**, MCM **(C, D)** and MCM + VB **(E, F)** animals. Fibrous tissue (ft), cartilage tissue (ct), native bone (borders marked by solid line), and regenerated bone (borders marked by dashed line) are indicated. Scale bars: 500 µm. **G, H**: Total periosteal callus area of control (n = 8), MCM (n = 8) and MCM + VB (n = 8) femurs at 14 days **(G)** and 70 days **(H)** after osteotomy. **I, J**: Callus composition including fibrous tissue (white), cartilaginous tissue (gray) and osseous tissue (black) of control (n = 8), MCM (n = 8) and MCM + VB (n = 8) femurs at 14 days **(I)** and 70 days **(J)** after osteotomy. Mean ± SEM; ^#^ *p* < 0.05 vs control; ^*^ *p* < 0.05 vs MCM.

The histomorphometric analysis on days 14 and 70 after osteotomy demonstrated that the total periosteal callus area of animals of the MCM + VB group was significantly larger than that of animals of the MCM and control group (Figure 5A–H). Intergroup comparisons showed no significant difference between the callus area of MCM animals compared to that of controls (Figure 5G,H).

Further evaluation of the callus composition revealed at 14 days after osteotomy a significantly increased fraction of osseous tissue and vice versa a reduced fraction of fibrous tissue in animals of the MCM + VB group (Figure 5A,C,E,I). This difference in callus composition between the three study groups could also be detected at 70 days after osteotomy (Figure 5B,D,F,J). These results indicate an endochondral bone formation process in the MCM + VB group, while the MCM group and controls revealed typical histological signs of atrophic non-unions.

Accordingly, the osseous bridging score after osteotomy was higher in animals of the MCM + VB group than that in animals of the MCM and control group at 14 days (MCM + VB: 2.8 ± 0.7; MCM: 0.0 ± 0.0; control: 0.0 ± 0.0; *p* < 0.05) as well as at 70 days (MCM + VB: 3.7 ± 0.3; MCM: 0.3 ± 0.3; control: 0.0 ± 0.0; *p* < 0.05).

### Immunohistochemical Analysis

**FIGURE 6:**
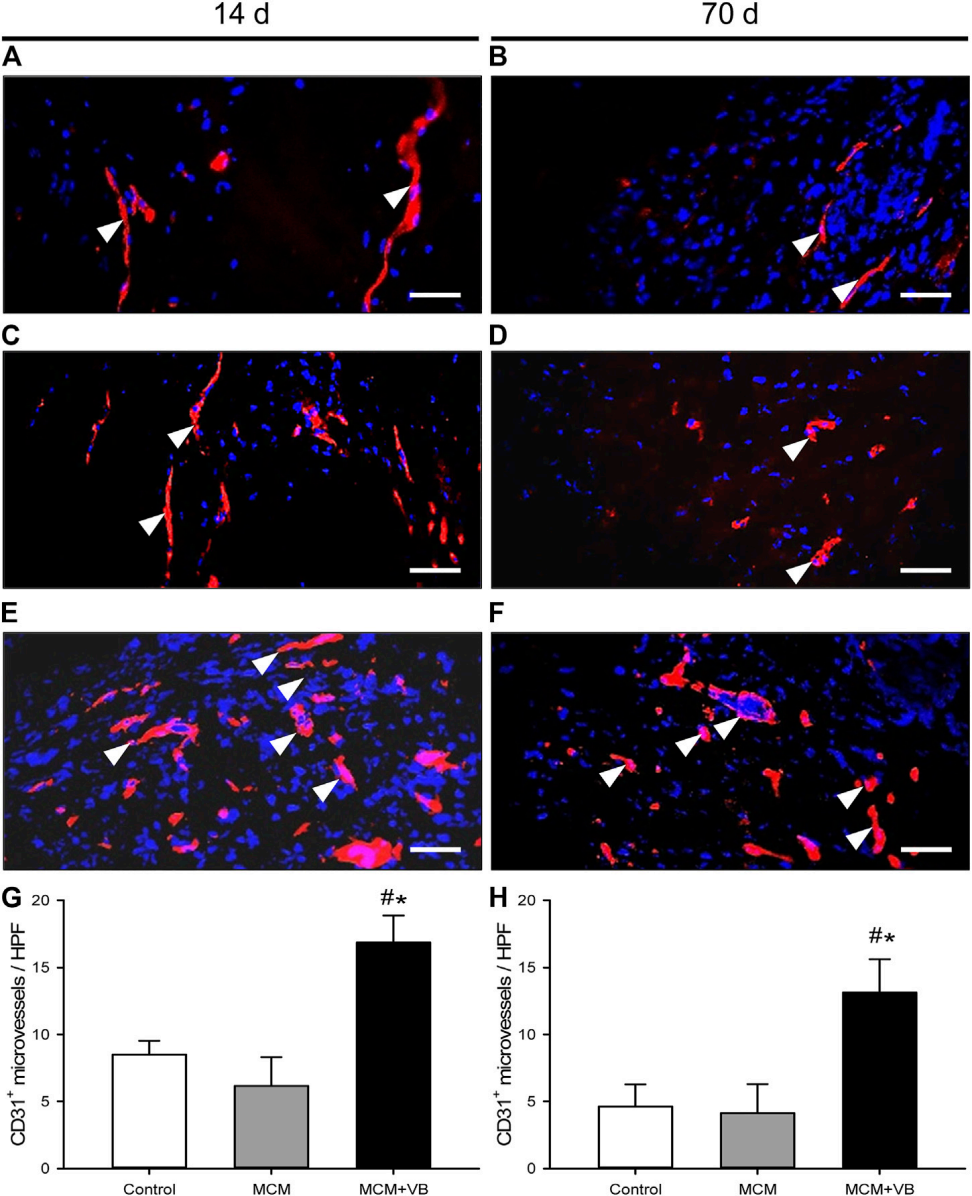
Immunohistochemical analysis of microvessels in the central healing zone of the periosteal callus. **A-F**: Immunohistochemical detection of CD31^+^ microvessels (arrowheads) within the callus tissue of osteotomized femurs of control **(A, B)**, MCM **(C, D)** and MCM + VB **(E, F)** animals at 14 days **(A, C, E)** and 70 days **(B, D, F)** after osteotomy. Bars represent 50 µm. **G, H:** Number of CD31^+^ microvessels per HPF of control (n = 4), MCM (n = 4) and MCM + VB (n = 4) femurs at 14 days **(G)** and 70 days **(H)** after osteotomy. Mean ± SEM; ^#^ *p* < 0.05 vs control; ^*^ *p* < 0.05 vs MCM.

The immunohistochemical detection of CD31^+^ microvessels in the periosteal callus at 14 and 70 days after surgery revealed significantly more vessels in MCM + VB animals when compared to MCM and control animals (Figure 6). This indicates that angiogenesis was improved in MCM + VB animals throughout the study period.

### Western Blot Analysis

**FIGURE 7:**
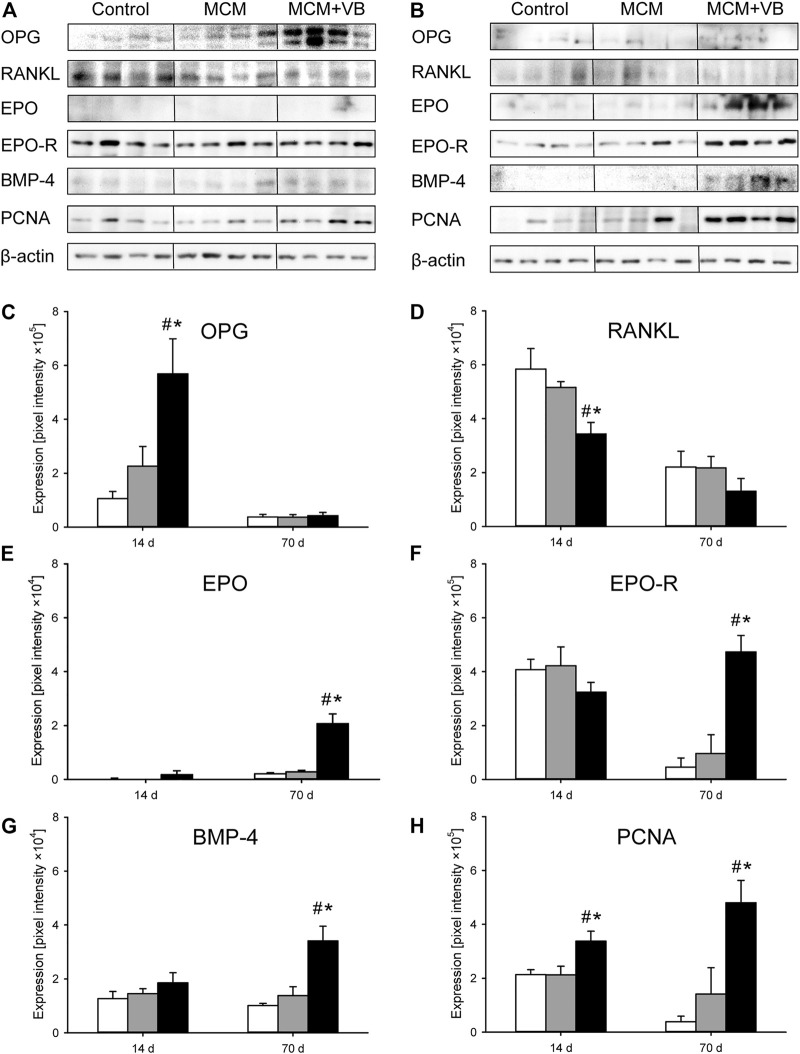
Western blot analysis of callus tissue. **A, B:** Representative Western blots of OPG, RANKL, EPO, EPO-R, BMP-4, PCNA and β-actin expression within the callus tissue of control, MCM and MCM + VB femurs at 14 days **(A)** and 70 days **(B)** after osteotomy. **C-H:** Expression of OPG **(C)**, RANKL **(D)**, EPO **(E)**, EPO-R **(F)**, BMP-4 **(G)** and PCNA **(H)** within the callus tissue of control (white; n = 4), MCM (gray; n = 4) and MCM + VB (black; n = 4) femurs at 14 and 70 days after osteotomy. Mean ± SEM; ^#^ *p* < 0.05 vs control; ^*^ *p* < 0.05 vs MCM.

The Western blot analysis revealed a significantly higher expression of OPG in callus tissue of MCM + VB when compared to callus tissue of MCM and control animals on day 14 after osteotomy (Figure 7A,C). On day 70 after surgery, no differences in expression of OPG between the groups could be observed (Figure 7B,C). The expression of RANKL was significantly reduced in the callus of MCM + VB animals when compared to that of MCM and controls at 14 days after surgery (Figure 7A,D), but did not show significant differences at 70 days (Figure 7B,D). The expression of EPO showed no differences between the study groups at day 14 after surgery (Figure 7A,E). In contrast, the expression of EPO increased in callus tissue of MCM + VB animals and showed an approximately 8-fold higher expression at day 70 after surgery when compared to callus tissue of MCM and control animals (Figure 7B,E). The expression of EPO-R did not show differences between the study groups at 14 days (Figure 7A,F). In contrast, its expression was significantly higher in the callus of MCM + VB animals compared to that of MCM and control animals at day 70 after surgery (Figure 7B,F). Similar to EPO, the expression of BMP-4 was not different between the groups on day 14 after surgery (Figure 7A,G), but demonstrated a significant increase in callus of MCM + VB animals at the late time point and thereby exhibited a significantly higher expression compared to callus tissue of MCM and controls (Figure 7B,G). The expression of the proliferation marker PCNA was increased in callus tissue of MCM + VB compared to MCM and controls at both time points (Figure 7A,B,H). These findings indicate an expression profile in animals of the groups MCM + VB typical for significantly reduced osteoclast activity at 14 days after surgery and improved vascularization and also enhanced osteogenic activity at 70 days after surgery.

## Discussion

The present study demonstrates for the first time the use of MCM as carriers for therapeutic delivery of VEGF and BMP-2 in atrophic non-unions. Our results confirm the hypothesis that the combination of VEGF- and BMP-2-loaded MCM are capable of improving bone repair in a well-established non-union model in mice. This was indicated by radiological signs of osseous bridging of the osteotomy, an improved biomechanical stiffness, an increased bone volume within the callus, including ongoing mineralization of the new bone during the study period, and a histologically larger total periosteal callus area. These effects can be explained by a reduced osteoclast activity at an early time point and a pro-angiogenic and pro-osteogenic protein expression profile at a late time point, as shown by Western blot analyses of the callus tissue. In fact, the resulting bone formation 70 days after surgery showed nearly full bone healing in MCM + VB animals, as indicated by radiological signs of remodeling, a callus composition consisting predominantly of osseous tissue and a high histological bridging score of the non-unions.

The delivery of growth factors and other bioactive agents by biocompatible carrier systems is difficult due to the susceptibility to denaturation and degradation as well as the heterogenous requirements during binding, cargo and release of the loaded proteins (Yu et al., 2017; Orth et al., 2019). We have developed MCM as a carrier for the controlled protein delivery. MCM were coated by a hydroxyapatite layer having nanostructured porosity (Yu et al., 2017). They have previously been shown to enable robust protein binding by electrostatic interactions between hydroxyapatite mineral surface and the side chains of the loaded proteins. Especially, the large surface area of MCM enables the efficient binding even of high amounts of the deliverable protein. We could further systematically modulate the physicochemical properties of mineral coating (e.g., pore size, porosity and dissolution rate) by varying the concentrations of ionic constituents or adding dopants in the mSBF used for the coating solution, which, in turn, dictates the release kinetics of delivered proteins for an extended time period (Lee et al., 2010; Lee et al., 2011; Suárez-González et al., 2012; Yu et al., 2014). In addition, the nanostructured mineral coating of MCM can enhance the stability of bound proteins against external stressors during formulation, storage and release, including organic solvents, proteases, and ethylene oxide gas sterilization (Yu et al., 2017). Accordingly, MCM allow the controllable binding and spatiotemporal controlled release of a defined quantity of growth factors, and have proved their therapeutic efficacy in various animal models (Yu et al., 2014; Dang et al., 2016; Dang et al., 2016b; Orth et al., 2017; Yu et al., 2017; Clements et al., 2018; Hellenbrand et al., 2019; Orth et al., 2019; Hellenbrand et al., 2021). In particular, our previous studies showed that MCM can bind VEGF and BMP-2 with high affinity and release them over 50 days in a sustained manner (Orth et al., 2017; Orth et al., 2019).

The modulation of bone healing to prevent or to treat non-unions represents a major challenge. In fact, the effect of additional application of VEGF to BMP-2 on bone healing is discussed controversially in the literature. It has recently been shown that the simultaneous or tunable co-delivery of low-dose BMP-2 and VEGF fails to fully restore the mechanics of bone in a bone defect model (Subbiah et al., 2020). Moreover, Samee et al. (2008) found that human periosteum-derived cells transfected with BMP-2 and VEGF *in vitro* do not show more bone formation at 8 weeks after implantation than human periosteum-derived cells transfected only with BMP-2. On the other hand, several studies could demonstrate that the co-application of VEGF and BMP-2 significantly improves bone regeneration when compared to single application of BMP-2 (Luo et al., 2012; Çakır-Özkan et al., 2017; Liu et al., 2020). The application of MCM loaded only with VEGF has been demonstrated to improve bone healing in a previous study using the identical murine non-union model (Orth et al., 2019). However, bone healing was still incomplete 70 days after surgery, as indicated by low values of bending stiffness, a low osseous bridging score and limited osseous tissue within the callus (Orth et al., 2019). The application of MCM loaded only with BMP-2 also resulted in improved bone healing with a high osseous bridging rate and increased osseous tissue fraction within the bone defects (Orth et al., 2017). However, the bending stiffness of the treated bones exhibited only 36% of that measured for unfractured femurs. Furthermore, there were no signs of bone remodeling in X-ray analyses, as observed in the present study. We have previously demonstrated that a hydrogel loaded with microvascular fragments as a potent angiogenic biomaterial may have beneficial effects on angiogenesis during the course of bone healing (Orth et al., 2018). However, we could show that sole support of angiogenesis may even lead to impaired bone healing (Orth et al., 2018). Therefore, the local application of a biomaterial to a non-union needs to respect the diverse interplay of all protagonists within the callus *in vivo* and should address both, angiogenesis and osteogenesis, in order to have a beneficial effect on the healing course (Orth et al., 2018; Orth et al., 2019; Muire et al., 2020). In line with this view, the combined application of VEGF and BMP-2 by MCM herein resulted in an improved bone healing and vascularization of the callus, as demonstrated by radiological signs of remodeling, high absolute and relative bending stiffness, a callus composition consisting predominantly of osseous tissue, and an increased number of microvessels in the callus tissue.

Based on these promising results, it may be speculated that the ratio of VEGF to BMP-2 applied to the defect site plays a crucial role for the healing process. Of interest, Peng et al. demonstrated that a lower ratio of VEGF to BMP-2 promotes bone regeneration (Peng et al., 2005). In line with these findings, we herein demonstrated that a ratio of VEGF to BMP-2 of 1:2 is beneficial for the healing of non-unions. Other studies analyzing the ratio of VEGF- to BMP-2-transfected adipose stem cells on bone healing reported that an even greater shift of 1:9 towards BMP-2 enhances osteogenesis and angiogenesis (Lee et al., 2019). Therefore, we feel that the ratio of VEGF to BMP-2 is of pivotal importance and should be analyzed in more detail in future studies to find an optimal stimulation of angiogenesis and osteogenesis for the treatment of non-unions.

Angiogenesis and osteogenesis are essential for a successful bone healing process and are tightly coupled (Kanczler and Oreffo, 2008; Rather et al., 2019). Angiogenesis is regulated by hypoxia-inducible transcription factors as a physiological response to hypoxic conditions. VEGF itself can act as a hypoxic-like signal during normoxia and serves to induce the expression of EPO (Bellomo et al., 2006; Orth et al., 2019). EPO is a cytokine that is known to improve endochondral ossification and mechanical strength via EPO-R signaling (Holstein et al., 2007). Moreover, VEGF has further direct osteoanabolic effects and induces a chemotactic response by binding on VEGF-receptor one on osteoblasts and serves to secrete osteoanabolic factors by stimulating endothelial cells (Wang et al., 1997; Bouletreau et al., 2002; Beamer et al., 2010). Among others, osteogenesis is further regulated by BMPs, which are well-known to exert an osteogenic effect (Cheng et al., 2003; Chen et al., 2004; Mandal et al., 2016). Of interest, the effect of BMPs and VEGF seems to be reciprocally reinforcing. According to previous studies, VEGF application has shown to potentiate the healing response of BMPs (Cui et al., 2013; Hankenson et al., 2015). BMP-4, in turn, increases the secretion of VEGF (Rivera et al., 2013). Of interest, Western blot analyses in the present study indicated that VEGF and BMP-2 upregulates pro-angiogenic EPO and EPO-R as well as pro-osteogenic BMP-4 at 70 days after surgery. Hence, it may be speculated that the application of MCM loaded with VEGF and BMP-2 synergistically stimulate angiogenesis and osteogenesis with respect to the events during the healing course and promote the substantiated positive effect on bone healing in this challenging non-union model.

Despite angiogenesis and osteogenesis, osteoclast activity is also important during the process of bone healing. As described previously, RANKL is a potent stimulator of bone resorption by binding receptor activator of NF-κB (RANK) in the cell membrane of osteoclasts (Boyce and Xing, 2008). It has been shown that an increased number of osteoclasts may impair bone healing due to a shift of the RANKL/OPG ratio within the callus during the early phase of bone healing (Orth et al., 2018). In contrast, in the present study the expression of OPG was increased, while the expression of RANKL was low at an early time point after surgery and, thus, shifting the ratio of RANKL to OPG towards OPG. As described elsewhere, this ratio is similarly modified with anti-RANKL treatment by OPG and leads to improved implant fixation and stability in bone (Bernhardsson et al., 2015). Moreover, the application of OPG as a systemic RANKL inhibitor together with BMP-2 enhances bone healing compared to BMP-2 treatment alone in a murine critical-sized femoral defect model (Bougioukli et al., 2016). In line with these findings, the shift of the RANKL/OPG ratio in the present study may have supported the osseous tissue formation by reducing the osteoclast activity during the early phase of bone healing.

In conclusion, the use of MCM for delivery of VEGF and BMP-2 shows a great potential to improve bone healing in atrophic non-unions. In the present study, the combined application of these angiogenic and osteogenic growth factors improved bone healing better than the application of one of these growth factors alone. We found that the ratio of VEGF to BMP-2 seems to be of pivotal importance and that a ratio of 1:2 locally applied to the defect site is beneficial for bone healing in non-unions. This result was most probably due to a synergistic effect of the applied angiogenic and osteogenic growth factors during the course of bone healing and a reduced osteoclast activity in the early phase of bone healing. Therefore, MCM as a carrier and spatiotemporal controlled release system for VEGF and BMP-2 should also be of interest for the treatment of delayed fracture healing and non-unions in clinical practice.

## Data Availability

The original contributions presented in the study are included in the article/supplementary material, further inquiries can be directed to the corresponding author.
